# Effect of Ambulance Stretcher Bed Height Adjustment on CPR Quality and Rescuer Fatigue in a Laboratory Environment

**DOI:** 10.7150/ijms.59037

**Published:** 2021-05-27

**Authors:** Chun-Sheng Ho, Yi-Ju Hsu, Fang Li, Chien-Ching Tang, Cheng-Pang Yang, Chi-Chang Huang, Chin-Shan Ho, Chun-Hao Chang

**Affiliations:** 1 Department of Physical Therapy, College of Medical and Health Science, Asia University, Taichung City, Taiwan.; 2 Division of Physical Medicine and Rehabilitation, Lo-Hsu Medical Foundation, Inc., Lotung Poh-Ai Hospital, Yilan City, Taiwan.; 3 Graduate Institute of Sports Science, National Taiwan Sport University, Taoyuan City, Taiwan.; 4 College of Exercise and Health Science, National Taiwan Sport University, Taoyuan City, Taiwan.; 5 First Corps, Fire Department, New Taipei City Government, New Taipei City, Taiwan.; 6 Department of Orthopedic Surgery, Chang Gung memorial hospital, Linkou Branch, Taoyuan City, Taiwan

**Keywords:** ambulance, stretcher bed, CPR quality, rescuer fatigue, chest compression

## Abstract

**Background:** The quality of cardiopulmonary resuscitation (CPR) is closely related to the survival rate of a patient, and it is crucial to maintain the quality of CPR during the ambulance journey to the receiving hospital. The purpose of this study was to investigate the effects of different stretcher bed heights on operator CPR quality.

**Methods:** In this randomized crossover trial, 16 male emergency medical technicians-paramedics (EMT-Ps) performed continuous chest compressions on a hemimorphic mannequin for 5 minutes, alternating between the current height of the stretcher bed on the ambulance (38 ± 1 cm) (S-38) and the height of the participant's midpoint of the patella (S-knee), where the stretcher bed surface is.

**Results:** According to the analysis of the quality of CPR exercises with two different stretcher bed heights at 5 minutes of continuous chest compression, the mean chest compression depth (CCD) of the S-38 position (53.81 ± 1.91 cm) was significantly lower than that of the S-knee (55.12 ± 2.03 cm; *p* < 0.001). The mean chest compression rate (CCR) of the S-38 position (111.44 ± 3.44 beats/min) was significantly higher than that of the S-knee (109.63 ± 4.46 beats/min; *p* = 0.027). The mean of total chest compressions (TCC) of the S-38 position (557.44 ± 16.81 times) was significantly higher than that of the S-knee (548.24 ± 19.40 times; *p* = 0.029). The rating of perceived exertion (RPE) of the S-38 position was significantly higher than that of the S-knee (12.75 ± 1.91 %; *p* = 0.015). Only the chest compression rebound rate (CCRR) (S-38: 97.56 ± 4.63 % vs. S-knee: 98.31 ± 1.89 %, *p* = 0.401) and the chest compression fraction (CCF) (S-38: 98.44 ± 0.81 % vs. S-knee: 98.44 ± 0.96 %, *p* = 1.000) did not reach a significant difference.

**Conclusion:** When a resuscitator is performing chest compressions in a standing position in an ambulance, the excessive downward leaning of the resuscitator's upper body affects CPR quality and increases fatigue. This study has verified that setting the stretcher bed of the ambulance at the knee height of the EMTs provides better CPR quality and lower fatigue.

## Introduction

The most critical condition for emergency medical services (EMS) patients is out-of-hospital cardiac arrest (OHCA). Among all EMS incidents in 2018, Taiwan had as many as 20,201 OHCA cases 1. The American Heart Association (AHA) states that for every minute of delay in resuscitation of a patient with cardiopulmonary arrest, the patient's chance of survival decreases by 10%. High-quality CPR can be life-saving when given to patients 2. Early high-quality CPR, rapid defibrillation, and good care after cardiac arrest are important factors in the survival of OHCA patients 3.

To save more lives, healthcare providers must be able to provide high-quality CPR. According to the official 2015 and 2020 AHA Guidelines for CPR and emergency cardiovascular care (ECC), the standard for high-quality CPR in adults is to perform chest compressions at a rate of 100-120 times per minute and to a depth of at least 50 mm but not more than 60 mm. The chest needs to rebound to its normal position after each compression, with minimal compression interruptions and avoidance of overinflation 2, 4.

For time-sensitive diseases such as acute coronary syndrome and stroke, it is important to transfer patients to hospitals as soon as possible for targeted treatment. However, one of the major problems in transporting OHCA patients to the hospital is to perform and maintain high-quality CPR during the transport 3. In Taiwan, the total emergency care time from emergency medical technicians (EMTs) reaching the patient to arrival at the hospital (pre-hospital time) is 16.64 minutes, and with only one EMT in the back to care for the patient on the way to the hospital, EMTs have to make a judgment call between quality and time of first aid 1. Performing chest compressions is a maneuver that demands a lot of physical stamina. Fatigue may also affect the accuracy of chest compression depth during prolonged chest compressions. In a test conducted by health science college students, it was found that the accuracy rate dropped from 52% at minute 1 to 39% at minute 5 5. It is difficult to perform high-quality CPR during transport, and chest compressions may even be susceptible to prolonged interruptions during heart rate analysis or defibrillation because of unexpected road conditions 6. Another factor that affects the quality of CPR is fatigue. According to a study by Ashton, McCluskey, Gwinnutt, & Keenan (2002), fatigue of the ambulance crew significantly affects the quality of rescue, with a gradual decrease in compression performance per minute over a 3-minute period of uninterrupted chest compressions 7. Thus, it is important to reduce the early onset of muscle fatigue associated with prolonged chest compressions, especially in ambulances, which can be unstable platforms for the performance of CPR.

The depth of CPR chest compressions has a significant effect on survival rates 8-11. Performing chest compressions in an ambulance is not as easy as kneeling on the floor to control the quality of CPR, and therefore it is the goal of academics and healthcare providers to improve the quality of resuscitation during transport. Restricted to the height of the stretcher bed in the ambulance (approximately 38 ± 1 cm), the EMT must perform chest compressions in a standing position, which can affect the depth of chest compressions. Previous literature has also addressed the effect of bed height on CPR quality and suggested that optimal chest compressions are achieved when the bed height is closer to the mid-thigh height 12, 13. However, raising the stretcher bed too high would tend to increase risks because the space in the ambulance is limited. Therefore, the purpose of this study was to explore the effects of two different heights, the height of the stretcher bed set in the current ambulance and the height of the midpoint of the CPR provider's patella, on the quality of CPR operation, as well as to compare the CPR quality and fatigue levels of the rescuers so as to recommend the optimal height of the stretcher bed in an ambulance for CPR operation.

## Methods

### Study design

This study was designed as a counterbalanced crossover experiment in a laboratory environment, in which well-trained firefighters performed CPR on Laerdal^®^ Little Anne QCPR Manikin (Laerdal Medical, Stavanger, Norway) at different stretcher bed heights to investigate the effects of different stretcher bed heights on CPR quality and fatigue levels. This study was approved by the Institutional Review Board of Fu Jen Catholic University (New Taipei City, Taiwan). Prior to the commencement of the experiment, all subjects were required to complete an informed consent from.

### Participants

This study recruited 16 volunteers, all of whom were active male firefighters aged 30 to 42 who had received 1,280 hours of EMT-P training, passed the EMT-P qualifying exam, and received 96 hours of refresher training every three years in accordance with the Regulations of Ambulance Technician Management. They had staffed ambulances for emergency medical care and provided first-aid service for more than three years. All the participants were right-handed. Participants with upper extremity impairments, spine-related disorders, cardiovascular disease, and other disorders were excluded from the study, as such conditions were judged unsuitable for participation. The anthropometric characteristics of the study population are listed in Table 1.

### CPR quality measurement

All the participants were required to complete two CPR quality tests at different stretcher heights in random order: the current stretcher bed height (38 ± 1 cm) (S-38) in the ambulance and the vertical height of the participant's midpoint of the patella from the floor (S-knee). The participants followed the chest compression technique described in the 2015 AHA Guidelines (compression depth of 50-60 mm, compression rate of 100-120 times per minute, uninterrupted, complete rebound of the chest) to perform 5 minutes of chest compressions on a Laerdal^®^ Little Anne QCPR Manikin. Once the participant stopped performing CPR, the Borg Rating of Perceived Exertion (RPE) scale was used to inquire about the participant's level of fatigue at the moment of completing the test. The participants were required to rest for at least 50 minutes between the tests, and no additional exercise training or heavy work was allowed during the rest period to prevent them from muscle fatigue or feeling tired.

### Data Analysis

All 16 participants completed the tests in this study. Data from the Laerdal^®^ Little Anne QCPR Manikin and the RPE were exported to Microsoft Excel (Excel version in Microsoft Office 2013 for Windows). Data from the Laerdal^®^ Little Anne QCPR Manikin were calculated using the SimPad PLUS device (Laerdal, Stavanger, Norway) with activated SkillReporter software. This study adopted chest compression quality parameters of chest compression fraction (CCF), chest compression depth (CCD), chest compression rate (CCR), total chest compressions (TCC), and chest compression rebound rate (CCRR), as well as the rating of perceived exertion (RPE).

### Statistical Analysis

All data are presented as means ± standard deviation (SD). This study used Paired Sample t tests to investigate differences in CPR quality between the two stretcher heights and calculated the percentage differences between the two tests for all CPR quality parameters. The statistical software IBM SPSS Statistics version 20 (IBM Corp., New York, NY, USA) was used for statistical analysis. The significance level was set to *p* < 0.05.

## Results

### The effects of different stretcher heights on CPR quality

**Figure 1:**
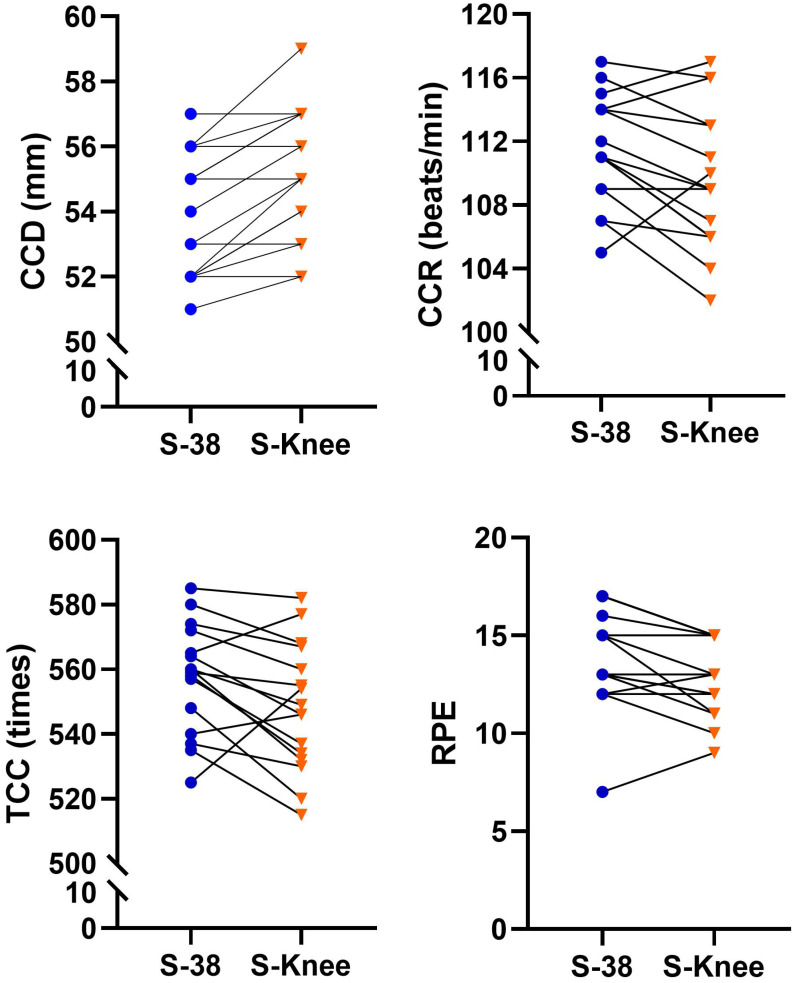
The performance of each EMT changes in CCD, CCR, TCC, and RPE scores of CPR.

Table 2 shows the differences in the CPR quality of chest compressions performed by the firefighters at two different stretcher bed heights. The quality of CPR performed at the two different stretcher bed heights was analyzed based on the criteria of a depth of 5 to 6 cm, a compression rate of 100 to 120 times per minute, and complete chest rebound. The paired sample t-test was used to analyze the effects of the two different stretcher bed heights on the CCD, CCR, CCRR, TCC, CCF, and RPE scores of CPR. The results indicated that the two different stretcher bed heights, S-38 vs. S-knee, yielded significant differences in CCD (S-38: 53.81 ± 1.91 mm vs. S-knee: 55.12 ± 2.03 mm; 0.27[95% CI, -1.89 to -0.74]; *p* < 0.001), CCR (S-38: 111.44 ± 3.44 beats/min vs. S-knee: 109.63 ± 4.46 beats/min; 0.74[95% CI, 0.24 to 3.38]; *p* = 0.027), TCC (S-38: 557.44 ± 16.81 times vs. S-knee: 548.24 ± 19.40 times; 3.87[95% CI, 1.08 to 17.29]; *p* = 0.029), and RPE (S-38: 13.75 ± 2.57 vs. S-knee: 12.75 ± 1.91; 0.37[95% CI, 0.22 to 1.78]; *p* = 0.015). Figure 1 shows the graph of the performance changes of each EMT in CCD, CCR, TCC, and RPE scores of CPR. Only CCRR (S-38: 97.56 ± 4.63 % vs. S-knee: 98.31 ± 1.89 %; 0.87[95% CI, -2.60 to 1.10]; *p* = 0.401) and CCF (S-38: 98.44 ± 0.81 % vs. S-knee: 98.44 ± 0.96 %; 0.20[95% CI, -0.44 to 0.44]; *p* = 1.000) were not found to have significant differences.

**Figure 2:**
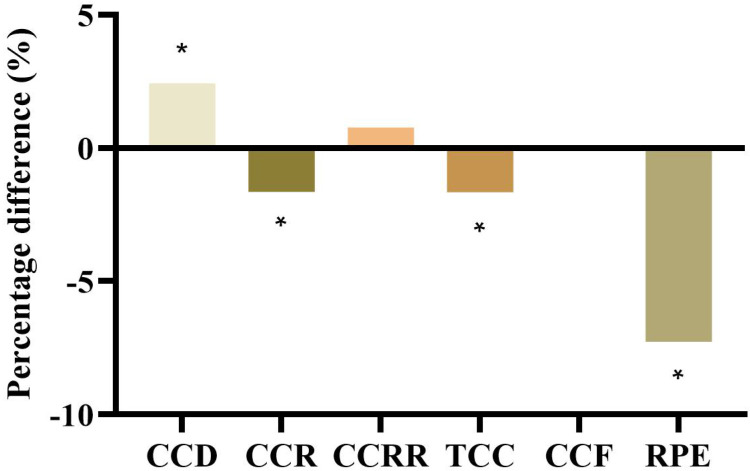
Percentage differences in CPR quality after adjustment of the stretcher bed height. *Indicates S-knee was significantly different from S-30, *p* < 0.05.

Figure 2 shows the degrees of improvement in CPR quality after the stretcher bed height was adjusted (from S-38 to S-knee). CCD increased by 2.43% (from 53.81 ± 1.91 mm to 55.12 ± 2.03 mm), CCR decreased by 1.64% (from 111.44 ± 3.44% to 109.63 ± 4.46%), CCRR increased by 0.77% (from 97.56 ± 4.63% to 98.31 ± 1.89%), TCC decreased by 1.65% (from 557.44 ± 16.81 times to 548.24 ± 19.40 times), CCF remained unchanged (from 98.44 ± 0.81% to 98.44 ± 0.96%), and RPE decreased by 7.27% (from 13.75 ± 2.57 to 12.75 ± 1.91).

## Discussion

Providing high-quality, consistent, and stable CPR in the ambulance is an important factor in determining patient survival. This study, where the height of the stretcher bed was adjusted in the limited space available, investigated and compared the effects of two stretcher bed heights on the quality of CPR when the CPR performer was in a standing position. Based on the AHA CPR Guidelines for 2015 and 2020 2, 4, the results of this study showed that all the participants were able to adapt to the different stretcher bed heights and perform high-quality CPR, with a CCF of 98% or higher (S-38: 98.44 ± 0.81%; S-knee: 98.44 ± 0.96%). We hypothesized that in the limited space of an ambulance, raising the stretcher bed height would maintain or improve the CPR quality of professional resuscitators as well as reduce physical fatigue during prolonged chest compressions. We found that S-knee, the stretcher bed height at the participant's knee height (S-knee: 47.3 ± 2.6 cm), maintained the CPR quality and reduced fatigue at the current stretcher bed height in the ambulance (S-38: 38 ± 1 cm) during prolonged chest compressions.

In a study by Foo, Chang, Lin, & Guo (2010), CPR was performed in different positions in the emergency room to compare the fatigue levels of healthcare workers performing CPR and investigate whether performing CPR would cause lower back pain. It was found that the overall accuracy rates of the 10-minute chest compressions were 73.2 ± 28% in the kneeling position, 67.4 ± 28% in the standing on the bench position, and only 59.1 ± 29% in the standing on the floor position 14. In a study by Mullin, Lydon, & O'Connor (2020), it was found that in an ambulance, the mean depth of chest compressions in a seated secured position (26 mm) was less than that in an unsecured standing position (52 mm). In addition, the unsecured standing position produced a higher percentage of correct compressions (83%) than did the seated secured position (8%). In contrast, the mean number of chest compressions and the chest compression rate had no evident differences. The participants also found chest compressions to be more effective when they were standing than when they were sitting. Thus, there is a strong correlation between the position of the rescuer in relation to the patient and the way the force is applied during CPR 15.

The fatigue of the rescuer during CPR affects the quality of the chest compressions. After the start of continuous chest compressions, the depth of chest compressions decreases over time due to the fatigue of the rescuer. Decreased depth of chest compressions decreases coronary perfusion pressure, which in turn decreases the likelihood of return of spontaneous circulation 16-18. Fatigue affects not only the depth of chest compressions but also other important parameters of CPR quality indicators. Past studies have suggested that faster chest compression rates are related to fatigue 7. As the chest compression rate increases, the chest rebound and compression depth scores worsen, and fatigue occurs sooner 20. We observed from the results of this study that the chest compression rate was significantly higher at the S-38 stretcher bed position than at the S-knee stretcher bed position. After the stretcher bed height was adjusted (from S-38 to S-knee), other CPR quality parameters improved, while CCF remained unchanged. For example, CCD increased by 2.43%, CCR decreased by 1.64%, TCC decreased by 1.65%, and RPE for fatigue level decreased by 7.27%.

The most effective bed height position is one in which the patient's or the mannequin's chest is in line with the CPR provider's mid-thigh, which allows the CPR provider to achieve the highest intrathoracic pressure during CPR 12, 13. In a study by Cho et al. (2009), it was found that the deepest chest compressions were achieved when the bed level was at the knee height of the CPR provider; comparatively, when the bed level was either above or below the knee of the CPR provider, there was a reduction in the chest compression depth. When the stretcher bed is positioned higher, however, the combination of poor technique and poor force generation results in up to a 22% reduction in the resultant intrathoracic pressure 12. The kinematic difference can be explained by the fact that when the patient's or the mannequin's chest is aligned with the CPR provider's mid-thigh, the resuscitator is able to straighten the arms and utilize the weight of the upper body to the fullest 13. On the other hand, when the stretcher bed is relatively too low relative to the rescuer's legs, the rescuer will experience excessive range of motion (ROM) in the lower back, resulting in increased lumbar stress. The rescuer will thus expend more energy to maintain high-quality compressions, leading to fatigue or early onset of muscle soreness, which can affect the quality of CPR 14. Thus, an appropriate stretcher bed height can maintain the quality of CPR and reduce the fatigue of the ambulance crew. Using the concept of an individualized, adjustable stretcher bed height, we adjusted the stretcher bed to the ambulance attendant's knee so that the mannequin's thorax was approximately aligned with the EMT's mid-thigh, which yielded results similar to those of previous studies.

The goal of this study was to determine how to improve the quality of ambulance services during transport. Currently, technological advances are leading to a gradual rise in ambulances equipped with chest compression machines in emergency ambulance services. Non-urban areas, however, tend to have uneven distribution of emergency-related equipment and personnel, or the inevitable, urgent situation where EMTs are unable to change hands and have to perform CPR as constantly as possible before the ambulance arrives at the hospital. Therefore, to reduce the fatigue caused by prolonged chest compressions and maintain the quality of CPR, it is necessary to establish and improve a user-friendly ambulance emergency space. This study has demonstrated that professional EMT-Ps who perform chest compressions can achieve better CPR quality and experience less fatigue when the stretcher bed is at the height of their knees than when it is at a height of 38 cm.

## Limitations

There are several limitations to this study. First, we did not include female EMT-Ps as participants. In this study, the average age of all the participants, who were representative of the majority of the local ambulance personnel population, was 34.3 ± 3.4 years (range: 30-42 years). Therefore, the results cannot be generalized to situations where female paramedics perform chest compressions. Second, this study was conducted in a simulated stationary ambulance in a laboratory setting, using a stretcher bed exclusively for the ambulance and a mannequin to test chest compressions by adjusting the bed height. The participants were not challenged by the acceleration, deceleration, and gravity inherent to moving ambulances 15, 21. Third, this study did not use objective electromyography (EMG) to measure the muscle fatigue of the participants; only the subjective RPE scale was used to understand the participants' perception of fatigue as an assessment of fatigue. Finally, our results were obtained using a mannequin and thus may differ from those obtained from human subjects. We hope to further discuss the feasibility of practical application and the survival rate of patients in the future.

## Conclusion

When a resuscitator is performing chest compressions in a standing position in the ambulance, the greater ROM of the resuscitator's lower back causes a greater impact on CPR quality and fatigue. High-quality CPR may not be maintained during prolonged transport, thus reducing the effectiveness of resuscitation. The objective of this study is to influence or improve overall CPR quality through slight adjustments in stretcher bed height. A large body of research indicates that the optimal bed height required to perform chest compressions is highly likely to be approximately the resuscitator's knee height. As the results of this study also indicate, setting the stretcher bed of an ambulance at the knee height of an EMT provides better CPR quality and lower fatigue. We expect this method to be applied to the user-friendly space configuration of the ambulance and at the same time to improve the work quality of the ambulance crew and maintain the CPR quality.

## Figures and Tables

**Table 1:** Anthropometry characteristics of participants

	Mean ± SD	Range
Age (years)	34.3 ± 3.4	30.0-42.0
Height (cm)	173.4 ± 5.0	165.0-183.0
Body mass (kg)	75.6 ± 8.8	67.0-95.0
BMI (kg/m^2^)	25.1 ± 2.8	21.4-31.2
knee height (cm)	47.3 ± 2.6	44.0-52.0
Seniority (years)	10.7 ± 4.0	6.0-20.0

Mean ± SD, mean value ± standard deviation. BMI, body mass index.

**Table 2:** Differences in CPR quality with different stretcher bed heights

	S-38	S-knee	t	*p*
CCD (mm)	53.81 ± 1.91	55.12 ± 2.03	4.869	< 0.001
CCR (beats/min)	111.44 ± 3.44	109.63 ± 4.46	-2.459	0.027
CCRR (%)	97.56 ± 4.63	98.31 ± 1.89	0.864	0.401
TCC (times)	557.44 ± 16.81	548.24 ± 19.40	-2.416	0.029
CCF (%)	98.44 ± 0.81	98.44 ± 0.96	0.000	1.000
RPE	13.75 ± 2.57	12.75 ± 1.91	-2.739	0.015

S-38, stretcher with a height of 38 cm. S-knee, stretcher at knee height. CCD, chest compression depth. CCR, chest compression rate. CCRR, chest compression rebound rate. TCC, total chest compressions. CCF, chest compression fraction. RPE, rating of perceived exertion.

## Abbreviations

CPR: cardiopulmonary resuscitation
EMT-Ps: emergency medical technicians-paramedics
CCD: chest compression depth
CCR: chest compression rate
TCC: total chest compressions
RPE: rating of perceived exertion
CCRR: chest compression rebound rate
CCF: chest compression fraction
EMS: emergency medical services
OHCA: out-of-hospital cardiac arrest
AHA: American Heart Association
ECC: emergency cardiovascular care
EMTs: emergency medical technicians
ROM: range of motion
EMG: electromyography
BMI: body mass index
